# Supplementary material for: Influence of Hydrophobic Side-Chain Length in Amphiphilic Gradient Copoly(2-oxazoline)s on the Therapeutics Loading, Stability, Cellular Uptake and Pharmacokinetics of Nano-Formulation with Curcumin
Source: Pharmaceutics. 2022 Nov 23;14(12):2576. doi: 10.3390/pharmaceutics14122576 (PMC9781838; doi:10.3390/pharmaceutics14122576)
Supplement: Supplementary file 1 [file pharmaceutics-14-02576-s001.zip › pharmaceutics-2026751-supplementary.pdf]

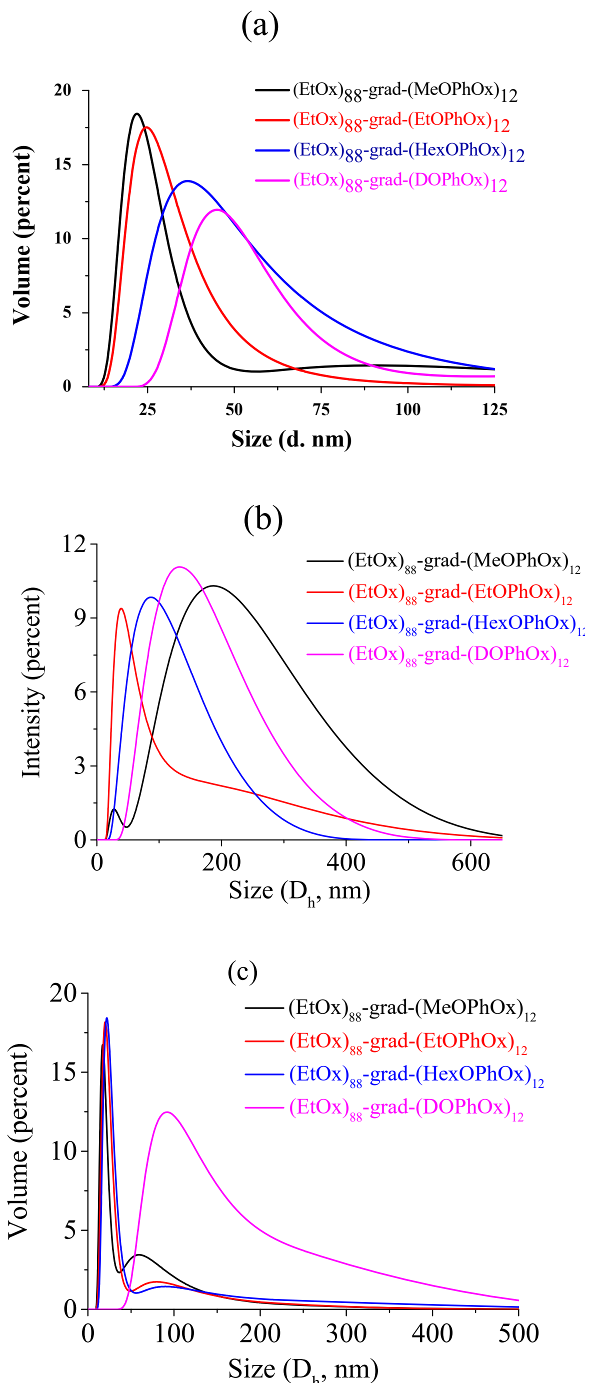

Figure S1. Volume weighted (a) and intensity weighted (b) size distribution plot of curcumin-loaded POx-NPs in PBS of pH 7.4 at 25 °C after 24 h from the time of their preparation. Volume weighted (c) size distribution plot at 96 h. The concentration of Polymeric NPs is kept at 800µg/mL. The feeding concentrations of polymer and curcumin used for the preparation of NPs were 1mg/mL and 0.036mg/mL.

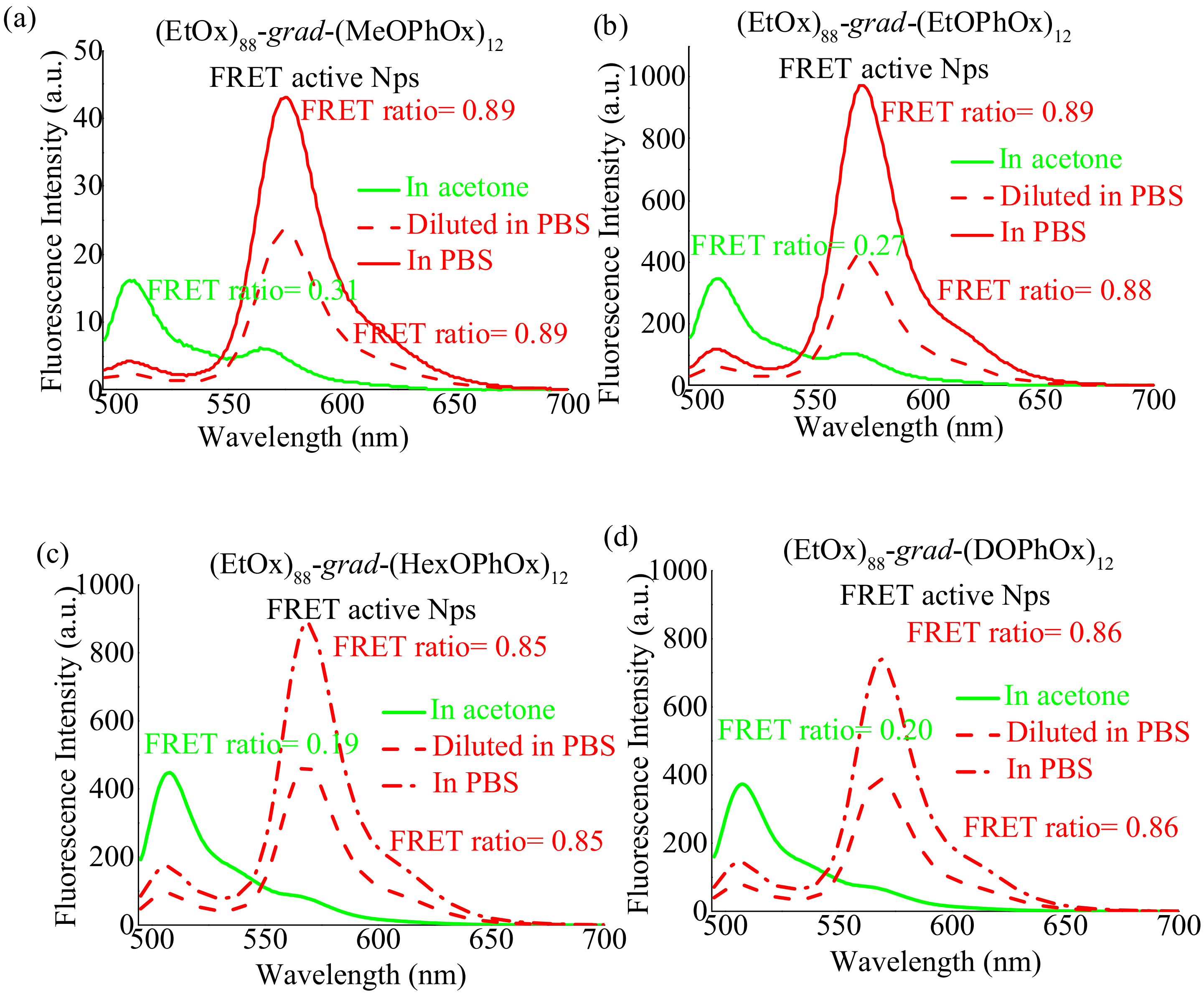

Figure S2. Steady-state fluorescence emission spectra of DiO (0.3wt %) and DiI (0.3wt %) co-loaded (a) (EtOx)-grad-(MeOPhOx), (b) (EtOx)-grad-(EtOPhOx), (c) (EtOx)-grad-(HexOPhOx), and (d) (EtOx)-grad-(DOPhOx) FRET active NPs in PBS (pH 7.4) and diluted with an excess volume of acetone. The concentration of all four FRET active NPs is kept at 800 μg/mL. The excitation wavelength is 484 nm and the emission spectra are recorded from 500-700 nm.

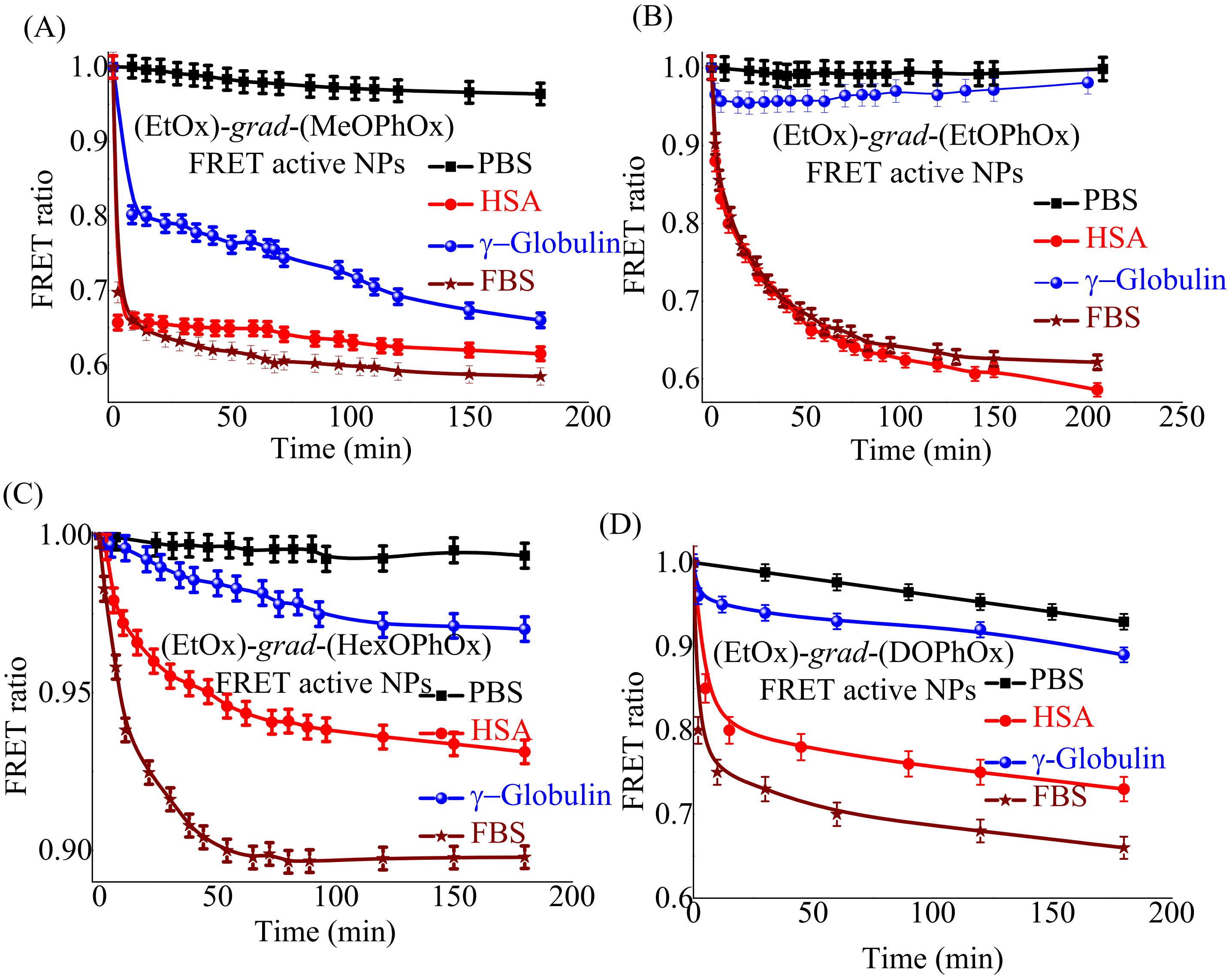

Figure S3. Variation of FRET ratio (Normalized to time zero) with time of (A) (EtOx)-*grad*-(MeOPhOx), (B) (EtOx)-*grad*-(EtOPhOx), (C) (EtOx)-*grad*-(HexOPhOx), and (D) (EtOx)-*grad*-(DOPhOx) FRET active NPs upon incubation with PBS (pH 7.4), or HSA (35 mg/mL), or γ-Globulin (15 mg/mL), or FBS (10%, v/v) solutions in PBS of pH 7.4. (n=3 independent measurements, mean ± standard deviation plotted).

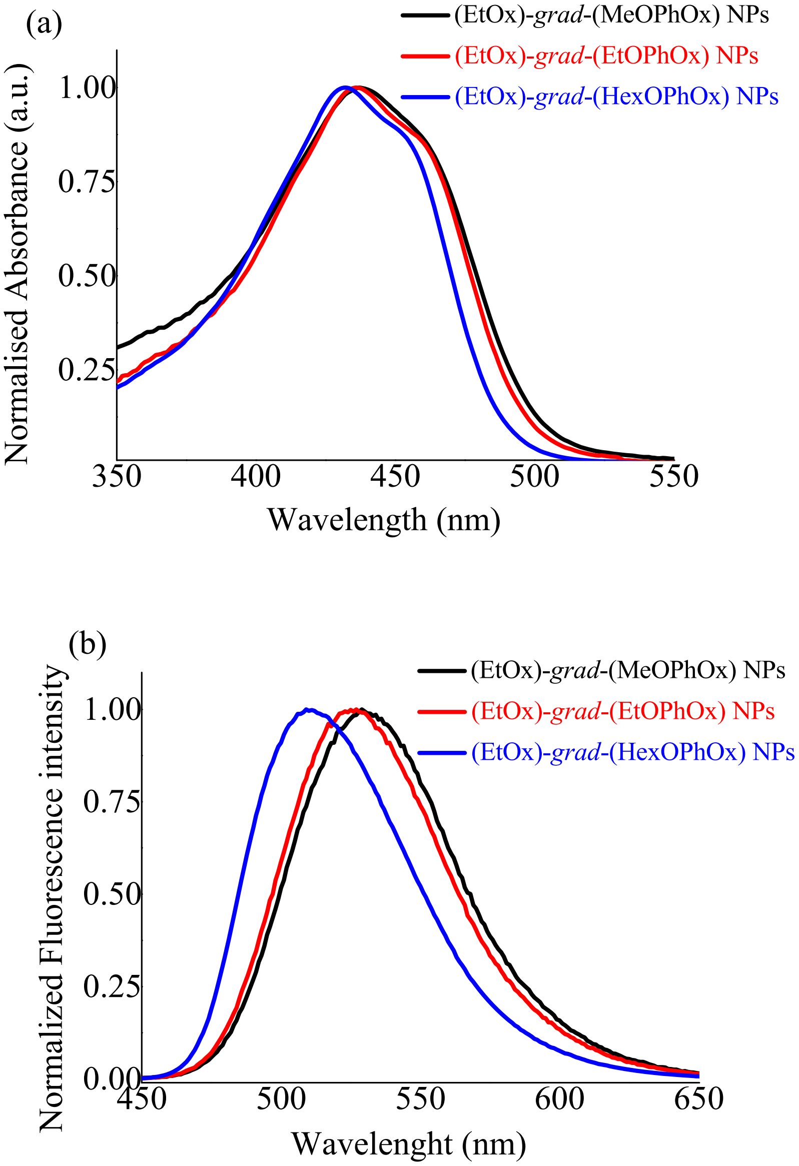

Figure S4. Normalised (a) absorbance spectra and (b) fluorescence emission spectra of curcumin-loaded POx NPs in PBS of pH 7.4.

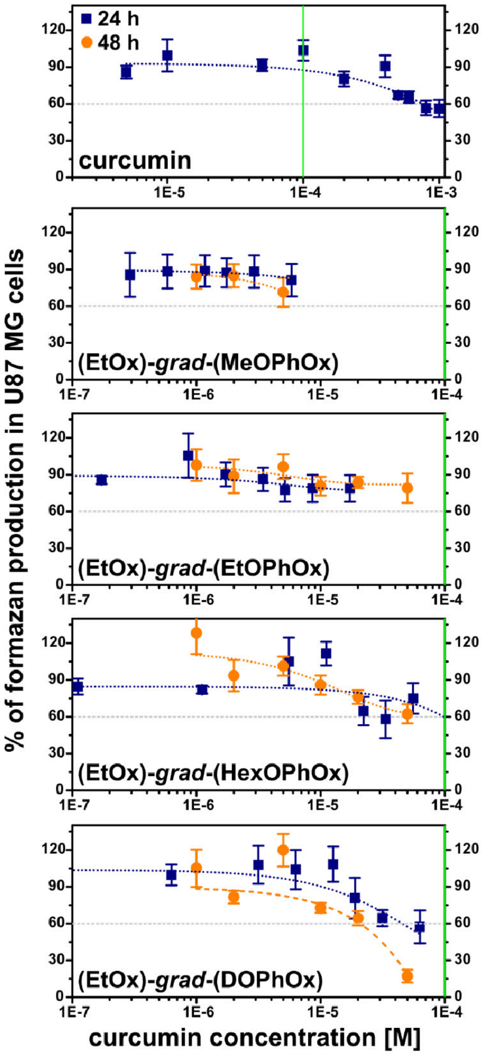

Figure S5. Metabolic activity of U87 MG cells in the presence of curcumin and curcumin- loaded polymeric nanoparticles (denoted in corresponding image) at concentration ranging between 10^{-7} -10^{-3} M. MTT test was performed 24 (blue squares) and 48 (orange circles) hours after compounds administration. Error bars represents standard deviations from the average (n = 16). Experimental data were fixed with exponential function (blue and orange lines). Green axes indicate the limit of 10^{-4} M that was not able to reach by particles preparation. Tested concentration for nanoparticles were below this value.

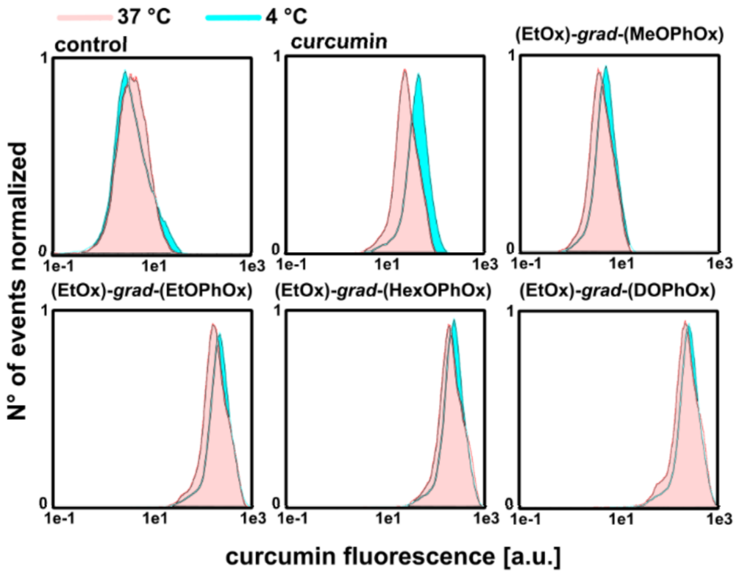

Figure S6. Uptake of curcumin and curcumin loaded polymeric NPs by U87 MG cells detected by flow cytometry. Fluorescence of curcumin was detected from U87 MG cells 1 h after administration and incubated at 37 °C (pink) and 4 °C (cyan).
